# Evaluation of vitamin D status and its correlation with oxidative stress markers in women with polycystic ovary syndrome

**Published:** 2017-06

**Authors:** Maryam Rahsepar, Soleiman Mahjoub, Sedigheh Esmaelzadeh, Maryam Kanafchian, Maryam Ghasemi

**Affiliations:** 1 *Student Research Committee, Babol University of Medical Sciences, Babol, Iran. *; 2 *Fatemeh Zahra Infertility and Reproductive Health Research Center, Babol University of Medical Sciences, Babol, Iran. *; 3 *Department of Clinical Biochemistry, Faculty of Medicine, Babol University of Medical Sciences, Babol, Iran. *

**Keywords:** Polycystic ovary syndrome, Vitamin D, Oxidative stress, Malondialdehyde

## Abstract

**Background:**

There is little evidence about antioxidant properties of vitamin D. Recent studies suggest that oxidative stress may play a major role in the pathophysiology of polycystic ovary syndrome (PCOS), but the association of vitamin D with oxidative stress is still not known in PCOS.

**Objective:**

The goal of the present study was to evaluate the correlation between serum 25-hydroxy vitamin D and oxidative stress markers in PCOS group compared to control group.

**Materials and Methods:**

60 PCOS women (20-40 yr old) and 90 healthy women as control group were participated in this case-control study. Fasting serum level of 25(OH) D, glucose, insulin, calcium, malondialdehyde (MDA), protein carbonyl (PC), also homeostasis model assessment for insulin resistance (HOMA-IR) and fasting glucose to insulin ratio (FGIR) were measured.

**Results:**

It was found that the mean of serum 25(OH)D was lower in the PCOS group (10.76±4.17) than in the control group (12.07±6.26) but this difference was not statistically significant (p=0.125). Fasting insulin, HOMA-IR and MDA were significantly higher in the PCOS patients as compared to the controls, whereas PC level did not differ for the two groups (p=0.156). No significant correlations were found between 25(OH)D levels and oxidative stress markers (MDA and PC).

**Conclusion:**

The findings indicated no significant differences in the serum 25(OH)D levels between the PCOS patients and the matched controls. Also, no correlation was found between the serum vitamin D levels and oxidative stress markers in both groups.

## Introduction

Polycystic ovary syndrome (PCOS) is one of the most frequent endocrinopathies among women at the reproductive age. The worldwide prevalence of this syndrome is 5-10% and it has been reported that 15.2% of Iranian women at the reproductive age are suffering from PCOS (1, 2). This syndrome is considered to be a heterogeneous abnormality with variable grades of reproductive and metabolic disorders. It seems that the interaction of genetic and environmental factors contributes to the pathogenesis of PCOS (3, 4). Insulin resistance is a common feature of this syndrome observed in 25-60% of women with PCOS (5). It seems that low levels of vitamin D might be a contributing factor in the pathogenesis of metabolic syndrome in PCOS (6). 

Recent evidence has indicated the associations between vitamin D levels and different PCOS symptoms, including insulin resistance, infertility and hirsutism (7). Abnormal circulating markers of oxidative stress are observed in women with PCOS independent of excess weight. This evidence suggests that oxidative stress may contribute to the pathophysiology of this syndrome (8).

Some studies have indicated that vitamin D may have antioxidant properties by modifying some antioxidant enzymes (9, 10). There are limited data about the association between 25(OH)D levels with oxidative stress markers and the effects of 25(OH)D on oxidative stress, while some studies have focused on type 2 diabetes (11, 12). However, there is no evidence about any associations between serum 25(OH)D levels and oxidative stress markers in women with PCOS.

The present research was conducted to evaluate the concentration of serum 25(OH)D and oxidative stress markers in PCOS and control groups. Also, the correlation between vitamin D and some oxidative stress markers was evaluated in women with PCOS as compared with healthy women.

## Materials and methods

**Participants**

This case-control study was done on 177 women, of whom 77 suffered from PCOS and 100 were healthy. The healthy ones formed the control group. All the women were 20-40 years old and referred to Fatemeh Zahra Infertility and Reproductive Health Research center, Babol, Iran, in 2015. Sample size with regard of α=0.05 and β=0.2 was determined based on previous studies by statistical consult (13, 14). The control and the patient groups were matched by age, weight and body mass index (BMI) variables. PCOS was diagnosed according to the revised Rotterdam criteria 2003 (15). Those with at least two of the following criteria were defined as PCOS patients: 

1. History of oligo- and/or anovulation 

2. Clinical and/or biochemical signs of hyperandrogenism 

3. Polycystic ovaries detected by ultrasound

Healthy women with regular menstrual cycles and with no evidence of polycystic ovary in their ultrasound, participated as the control group. All the participants were living in Mazandaran and Golestan, provinces in the north of Iran and had a no history of smoking or drug abuse.

The exclusion criteria were the presence of diseases such as diabetes mellitus, thyroid dysfunction, renal and liver disorders, and endocrine disorder with a similar clinical presentation such as congenital adrenal hyperplasia, Cushing’s syndrome, androgen-secreting tumors and hyperprolactinemia. Also, the subjects who had taken oral contraceptives, insulin sensitizing and fertility medication, antioxidants, vitamin D and calcium supplementation within three months prior to the study were excluded. The medical history of all the subjects was assessed based on their medical files. All the samples were collected during fall and winter.

**Biochemical measurements**

After a 10-12 hr overnight fasting, 5 ml blood samples were obtained and centrifuged at 3000 rpm for 20 min. Then, the serum samples were stored at -80°C. With all samples collected, vitamin D and the other parameters were measured. Fasting serum glucose (normal range= 75-110 mg/dl) was measured using the glucose-oxidase colorimetric method with a commercial kit (Pars Azmoon, Iran). Serum calcium concentration (normal range= 8.5-10.8 mg/dl) was measured using commercial kit (Darman Faraz Kave, Iran). Fasting serum insulin (normal range= 2-25 μIU/ml) was measured with an available commercial ELISA kit (Catalog No.DE2935; Demeditec Diagnostics GmbH, Germany). Also serum 25(OH)D levels (ng/ml) were measured using a 25-hydroxy vitamin D EIA kit, (Catalog No.AC-57SF1; IDS, UK). 

Malondialdehyde (MDA) is a common marker of lipid peroxidation. Its concentration in serum samples was measured by the TBARS (thiobarbituric acid reactive-substances) assay with spectrophotometry at 535 nm. The serum concentration of MDA was reported nmol/ml (16). Protein carbonyl (PC) is one of the biomarkers of oxidative stress of proteins in biological sample. It was measured by spectrophotometry at 370 nm using the methods devised by of Levine *et al* (17). The total protein of the samples was measured with the biuret method and by using a commercial kit of total protein (ZIESTCHEM.CO, Iran). Also, the carbonyl content of the samples was reported as nmol/mg protein.

The homeostatic model assessment for insulin resistance (HOMA-IR) was calculated as fasting glucose (mg/dl) × fasting insulin (μ IU/ml)/405. Also, calculation was made of the fasting glucose to insulin ratio (FGIR) (18). Serum vitamin D levels were described as deficient (<10 ng/ml), insufficient (10-29 ng/ml) and sufficient (30-100 ng/ml) (19).

**Ethical consideration**

This study was approved by the Ethics Committee of Babol University of Medical Sciences (Protocol No. MUBABOL.REC.1394.180) and informed consent was obtained from all participants of the study.

**Statistical analysis**

The data obtained from the experiments were analyzed using version 18 of the SPSS software program. Histograms and Kolmogorov-Smirnov method were used to estimate the data distribution. To analyze the differences between the two groups, a student’s t test for normally distributed data and a nonparametric Mann-Whitney U-test for abnormally distributed data were used. The correlations between the mean of 25(OH) D and other variables were analyzed by the Spearman correlation test. The results were expressed as (mean±SD) and the statistical significant was considered as p-value<0.05. 

## Results

According to the inclusion criteria, 177 participants were recruited in this study. Of them, 27 women were subsequently excluded from the analysis due to hyperprolactinemia (n=3), hypothyroidism (n=5), consumptions of fertility medicines (n=6), metformin (n=6) or lack of data in their records (n=7).

The demographic, biochemical and oxidative stress parameters of 150 valid subjects (60 PCOS patients and 90 healthy controls) are presented in Table I. The two groups were similar in term of demographic parameters (i.e. age, weight and BMI). The findings from all subjects indicated that, out 150 women, 76 (50.67%) had vitamin D deficiency, 69 (46%) had vitamin D insufficiency but only 5 women (3.33%) had a sufficient level. Among the PCOS subjects, 39 women (65%) had a deficient level and 21 (35%) had an insufficient level. 

Among the healthy subjects, there were 37 women (41.1%) with a deficiency, 48 (53.3 %) with an insufficiency and only 5 (5.6%) with a sufficient level. The mean of serum vitamin D was higher in the control group but there were no statistically significant differences between the two groups in this regard. (10.76±4.17 ng/ml in the patients vs. 12.07±6.26 ng/ml in the controls, p=0.125). There was a significant difference of fasting insulin, HOMA-IR, FGIR and MDA between the patients and the controls. Fasting insulin, HOMA-IR and MDA were significantly higher in the PCOS group than in the control group whereas FGIR was lower in the PCOS women. The PC level did not differ between the two groups (p=0.156)

All the PCOS women had hypovitaminos D (<30 ng/ml). The PCOS subjects were divided into two sub-groups including vitamin D deficiency (<10 ng/ml) and vitamin D insufficiency (10-29 ng/ml). Then, the PCOS subgroups were compared in terms of the oxidative stress markers. There were no statistically significant differences between the two PCOS subgroups (Table II). Due to the high percentage of vitamin D deficiency in the control group, subjects with vitamin D deficiency in this group were excluded and only the healthy subjects without vitamin D deficiency were considered as controls (n=53). The correlation between vitamin D and oxidative stress markers in both groups is shown in Table III. As it can be seen, no significant correlation was found between 25(OH) D levels and oxidative stress markers such as MDA and PC in both groups.

**Table I:** Demographic, biochemical and oxidative stress parameters of the PCOS and control subjects

**variable**	**PCOS (n=60)**	**Control (n=90)**	**p-value**
Age (year)	28.68 ± 5.08	29.17 ± 5.03	0.628^b^
Weight (kg)	71.05 ± 14.49	70.98 ± 11.98	0.974^a^
BMI (kg/m^2^)	29.14 ± 5.54	27.92 ± 4.70	0.150 a
25(OH)D (ng/ml)	10.76 ± 4.17	12.07 ± 6.26	0.125 b
Ca (mg/dl)	9.55 ± 1.95	9.16 ± 0.83	0.154 a
FBS (mg/dl)	85.90 ± 18.99	84.26 ± 15.20	0.560 a
Insulin (μIU/ml)	15.67 ± 7.88	11.80 ± 5.12	0.003 b
HOMA-IR	3.44 ± 2.11	2.46 ± 1.15	0.008 b
FGIR	7.05 ± 4.77	8.63 ± 4.36	0.003 b
MDA (nmol/ml)	3.12 ± 0.66	2.41 ± 0.56	<0.001 a
PC (nmol/mg protein)	0.324 ± 0.13	0.295 ± 0.09	0.156 a

Data are presented as mean ± SD and analyzed by student’s t test and Mann-Whitney U- test. Values are significant at P<0.05.

BMI: body mass index

25(OH) D: 25 hydroxy vitamin D

Ca: Calcium.

FBS: fasting blood sugar

FGIR: fasting glucose to insulin ratio

MDA: malondialdehyde,

PC: protein carbonyl

HOMA-IR: homeostasis model assessment of insulin resistance.

a Student’s t-test

b Mann-Whitney U test

**Table II:** Oxidative stress markers of the PCOS subjects based on 25 (OH)D status

**Oxidative stress markers**	**25(OH) D** **<10 ng/ml (n=39)**	**25(OH) D** **10-29 ng/ml (n=21)**	**p-value**
MDA (nmol/ml)	3.17 ± 0.70	3.04 ± 0.59	0.478
PC (nmol/mg protein)	0.360 ± 0.137	0.304 ± 0.130	0.125

Data are presented as mean ± SD and analyzed by student’s t test. The values are significant at P<0.05.

Deficient level (<10 ng/ml), Insufficient level (10-29 ng/ml)

MDA: malondialdehyde

PC: protein carbonyl

**Table III:** The correlation between 25 (OH) D and oxidative stress makers in the PCOS and control subjects

**Variable**	**PCOS (n=60)**		**control (n=53)**	
	**Spearman correlation coefficient**	**P-value**	**Spearman correlation coefficient**	**p-value**
MDA(nmol/ml)	-0.019	0.887	-0.105	0.456
PC (nmol/mg protein)	0.180	0.169	-0.063	0.653

Values are significant at P<0.05.

MDA: malondialdehyde

PC: protein carbonyl

## Discussion

The present study was designed to evaluate serum 25(OH)D levels and their correlation with oxidative stress markers in PCOS women. According to the findings of this study, 65% of PCOS women and 41.1% of healthy women suffer from vitamin D deficiency. In agreement with previous studies, a comparison of 25(OH)D levels showed no significant difference between the two groups, whereas Ghadimi *et al*, reported significantly lower vitamin D in PCOS girls (16-20 yr old) as compared to non-PCOS girls (13, 14, 20, 21). 

Vitamin D deficiency is a global health problem and 60-80% of Iranian women at reproductive age are vitamin D deficient (22). The possible causes for the high prevalence of vitamin D deficiency among Iranian women are sun light avoidance, skin pigmentation, insufficient intake of vitamin D in the Iranian diet and specific polymorphism in vitamin D receptor and vitamin D-binding protein (11). This study showed that PCOS women have a significantly high level of fasting insulin and HOMA-R when compared to healthy women.

Similar values are observed in other studies (21, 23, 24). Recent evidence indicates that genetic variation in the vitamin D receptor may affect PCOS development as well as insulin resistance in women with PCOS (25). Insulin resistance can augment oxidative stress because of hyperglycemia and higher levels of free fatty acids which lead to ROS generation. Oxidative stress can damage biological molecules and lead to dysfunction and death of cells (26). MDA is one of the stable markers of lipid peroxidation, which has detrimental effects on the cells. It was found that it is significantly higher in PCOS women as compared with healthy women and the obtained results are similar to those in previous studies (27-29). Also Murri *et al* performed a meta-analysis in this regard including 1481 women (790 women with PCOS and 691 controls). The mean MDA levels were increased by 47% in women with PCOS as compared with the controls, suggesting that MDA level might be one of best markers to represent the oxidative stress status in PCOS (8).

Protein carbonyls have been studied less than other oxidative markers in PCOS patients. In the present study, the increase of serum protein carbonyl level in women with PCOS was not significant. A significant increase of protein carbonyl in obese and non-obese PCOS women was reported in previous studies (30-32). All the PCOS subjects suffered from hypovitaminos D and the mean value of oxidative stress markers was higher in the PCOS women with vitamin D deficiency than the PCOS women with vitamin D insufficiency. However, there were no statistically significant differences between the two mentioned subgroups. In this respect, there are no previous reports about comparing of oxidative stress markers in PCOS subjects based on their vitamin D status. 

The new finding of this study regards the correlation between serum levels of 25(OH) D and oxidative stress markers in the PCOS patients, and no significant correlations was found between 25(OH)D levels with oxidative stress markers. For the first time Saedisomeolia *et al* evaluated the association between serum vitamin D and antioxidants markers in diabetic patients. They showed that vitamin D may have some effects on the control of oxidative stress in such patients (11). 

It should be noted that there have been only a few interventional studies about the effect of vitamin D supplementation on oxidative stress in which some oxidative stress markers significantly are decreased but some others have no change (12, 33, 34). The inhibitory effect of vitamin D against oxidative stress is not clear although it may be related to vitamin D receptor (VDR). In clinical experiment, hemodialysis patients were treated with paricalcitol, a selective VDR activator. A significant reduction of PC and MDA was observed after a three months treatment with paricalcitol (35). Vitamin D may influence oxidative stress through its effects on immune functions. Sardar *et al* conducted a study on diabetic rats and concluded that vitamin D decreases oxidative stress by up-regulating antioxidant enzymes such as superoxide dismutase (SOD) (36). Cytokines have a regulatory influence on circulating SOD and vitamin D can up-regulate superoxide dismutase through regulation of cytokines (34, 37).

**Limitation**

The limitations of the present study are as follows: First, The sample size was small, and it could decrease the reliability of the study to evaluate any possible correlation between vitamin D and oxidative stress markers. Second, the study evaluated the correlation between vitamin D and only two oxidative stress markers (i.e. MDA, PC). Evaluation of other oxidative stress markers and antioxidant enzymes as well may increase the significance and validity of such studies. Third, there was a high percentage of vitamin D deficiency among the participants, which seems to have affected the results of the study. 

## Conclusion

As it emerged in this research, low levels of vitamin D are prevalent in women specially those suffering from PCOS, but these differences is not statically significant. The study revealed that there is increased oxidative stress in women with PCOS but there is no correlation between vitamin D and oxidative stress markers in this group. Therefore, to evaluate the association between vitamin D and oxidative stress markers in PCOS patients further studies are required. In particular, interventional studies are recommended to evaluate the effects of vitamin D therapy on oxidative stress in PCOS women.

## Note

***This article extracted from M.Sc. thesis. (Maryam Rahsepar)***
